# Supplementary material for: Validating a cassava production spatial disaggregation model in sub-Saharan Africa
Source: PLoS One. 2024 Nov 5;19(11):e0312734. doi: 10.1371/journal.pone.0312734 (PMC11537372; doi:10.1371/journal.pone.0312734)
Supplement: S1 Appendix — (DOCX) [file pone.0312734.s001.docx]

## **Supporting information - Validating a cassava spatial disaggregation model in sub-Saharan Africa**

AOI 10.1088/1748-9326/acc476 briefly reviews AI/EO crop mappingA

BOI 10.1088/1748-9326/acc476 briefly reviews AI/EO crop mappingA

**S1 Fig.** Area under monoculture (A) or intercropped (B) cassava production at each survey location in Uganda.

**S2 Fig.** Fitted smooth functions to the models with the best AIC scores. The top panel represents results for survey data from Côte d'Ivoire with the top-left showing the 2-dimensional smooth fitted between CassavaMap production and population density whilst the top-right shows the 1-dimensional smooth fitted to the settlement density. The bottom panel represents results from Uganda with bottom-left showing the 2-dimensional smooth fitted between CassavaMap harvest area and population density and bottom-right showing the 1-dimensional smooth fitted to the settlement density.

**S1 Table.** List of all possible covariates extracted from raster layers associated with CassavaMap predictions, population densities and settlement densities respectively. Extracted covariates are different summary statistics of a buffered region at 2, 5 and 10km about each survey point. Only one term from each column is included in the regression modelling at a time*.*

| **CassavaMap predictions** | | |  | **Population summaries** | |  | **Settlement summaries** | |
| --- | --- | --- | --- | --- | --- | --- | --- | --- |
| **Prediction Type** | **Buffer Distance (km)** | **Summary Statistic** |  | **Buffer Distance (km)** | **Summary Statistic** |  | **Buffer Distance (km)** | **Summary Statistic** |
| Production | 0 | Point prediction |  | 0 | Point prediction |  | 2 | mean |
| Production | 2 | mean |  | 2 | mean |  | 5 | mean |
| Production | 5 | mean |  | 5 | mean |  | 10 | mean |
| Production | 10 | mean |  | 10 | mean |  |  |  |
| Production | 2 | sd |  | 2 | sd |  |  |  |
| Production | 5 | sd |  | 5 | sd |  |  |  |
| Production | 10 | sd |  | 10 | sd |  |  |  |
| Production | 2 | minimum |  | 2 | minimum |  |  |  |
| Production | 5 | minimum |  | 2 | lower quartile |  |  |  |
| Production | 2 | lower quartile |  | 5 | lower quartile |  |  |  |
| Production | 5 | lower quartile |  | 10 | lower quartile |  |  |  |
| Production | 10 | lower quartile |  | 2 | median |  |  |  |
| Production | 2 | median |  | 5 | median |  |  |  |
| Production | 5 | median |  | 10 | median |  |  |  |
| Production | 10 | median |  | 2 | upper quartile |  |  |  |
| Production | 2 | upper quartile |  | 5 | upper quartile |  |  |  |
| Production | 5 | upper quartile |  | 10 | upper quartile |  |  |  |
| Production | 10 | upper quartile |  | 2 | maximum |  |  |  |
| Production | 2 | maximum |  | 5 | maximum |  |  |  |
| Production | 5 | maximum |  | 10 | maximum |  |  |  |
| Production | 10 | maximum |  |  |  |  |  |  |
| Harvest Area | 0 | Point prediction |  |  |  |  |  |  |
| Harvest Area | 2 | mean |  |  |  |  |  |  |
| Harvest Area | 5 | mean |  |  |  |  |  |  |
| Harvest Area | 10 | mean |  |  |  |  |  |  |
| Harvest Area | 2 | sd |  |  |  |  |  |  |
| Harvest Area | 5 | sd |  |  |  |  |  |  |
| Harvest Area | 10 | sd |  |  |  |  |  |  |
| Harvest Area | 2 | minimum |  |  |  |  |  |  |
| Harvest Area | 5 | minimum |  |  |  |  |  |  |
| Harvest Area | 2 | lower quartile |  |  |  |  |  |  |
| Harvest Area | 5 | lower quartile |  |  |  |  |  |  |
| Harvest Area | 10 | lower quartile |  |  |  |  |  |  |
| Harvest Area | 2 | median |  |  |  |  |  |  |
| Harvest Area | 5 | median |  |  |  |  |  |  |
| Harvest Area | 10 | median |  |  |  |  |  |  |
| Harvest Area | 2 | upper quartile |  |  |  |  |  |  |
| Harvest Area | 5 | upper quartile |  |  |  |  |  |  |
| Harvest Area | 10 | upper quartile |  |  |  |  |  |  |
| Harvest Area | 2 | maximum |  |  |  |  |  |  |
| Harvest Area | 5 | maximum |  |  |  |  |  |  |
| Harvest Area | 10 | maximum |  |  |  |  |  |  |

**S2 Table.** ANOVA results from analysing cassava production variables against distinct administrative zones in Côte d’Ivoire.

| Response variable | Transformation | NDF, DDF | F value | p-value |
| --- | --- | --- | --- | --- |
| total area under cassava production | square root | 8, 60 | 1.88 | 0.079 |
| total area under cassava monoculture | square root | 8, 60 | 1.83 | 0.090 |
| total area under cassava intercropping | square root | 8, 60 | 1.44 | 0.200 |

**S3 Table.** ANOVA results from analysing cassava production variables across distinct administrative regions in Uganda.

| Response variable | Transformation | NDF, DDF | F value | p-value |
| --- | --- | --- | --- | --- |
| total area under cassava production | square root | 3, 83 | 1.83 | 0.147 |
| total area under cassava monoculture | square root | 3, 83 | 1.05 | 0.375 |
| total area under cassava intercropping | square root | 3, 83 | 2.62 | 0.056 |

**S4 Table.** Type-II ANOVA tables for the AIC and adjusted R^2^ extracted from all individual models fitted to Côte d'Ivoire. Only F-statistics are shown, with 31026 residual degrees of freedom, most terms greater than 1 are significant.

|  |  | **Total cassava area** | | **Total monoculture area** | | **Total intercropped area** | | **Total cassava area (weighted)** | | **Total monoculture area (weighted)** | | **Total intercropped area (weighted)** | |
| --- | --- | --- | --- | --- | --- | --- | --- | --- | --- | --- | --- | --- | --- |
| **Term** | **DF** | **F (AIC)** | **F (adj. R2)** | **F (AIC)** | **F (adj. R2)** | **F (AIC)** | **F (adj. R2)** | **F (AIC)** | **F (adj. R2)** | **F (AIC)** | **F (adj. R2)** | **F (AIC)** | **F (adj. R2)** |
| modeltype | 1 | 8061.29 | 15104.36 | 24732.70 | 50011.95 | 1058.44 | 1656.37 | 17717.25 | 31902.95 | 37548.20 | 63331.49 | 3083.09 | 5123.30 |
| cass_type | 1 | 0.51 | 10.78 | 774.36 | 508.57 | 102.12 | 351.62 | 161.84 | 89.00 | 842.81 | 427.06 | 82.24 | 164.25 |
| population_type | 1 | 799.62 | 134.20 | 16.17 | 0.54 | 1306.56 | 697.07 | 202.62 | 34.98 | 6.10 | 0.25 | 877.43 | 399.75 |
| settlement_type | 1 | 1597.37 | 5.15 | 35.18 | 0.22 | 4465.05 | 1588.73 | 300.54 | 0.79 | 1.36 | 5.16 | 3099.07 | 1090.27 |
| cass_dist | 3 | 875.69 | 1186.70 | 640.50 | 1593.68 | 345.18 | 779.12 | 1005.23 | 2200.74 | 877.72 | 1986.13 | 213.02 | 834.79 |
| cass_summary | 6 | 1962.28 | 2010.12 | 632.37 | 1100.35 | 422.96 | 322.85 | 1847.01 | 2672.92 | 1010.00 | 1736.01 | 339.13 | 308.56 |
| cass_type:population_type | 1 | 2.81 | 3.27 | 0.23 | 0.21 | 0.41 | 0.21 | 0.32 | 0.50 | 0.78 | 0.13 | 0.85 | 1.15 |
| cass_type:settlement_type | 1 | 1.23 | 5.73 | 9.05 | 0.01 | 12.34 | 28.11 | 0.37 | 11.13 | 4.80 | 0.66 | 8.96 | 17.21 |
| population_type:settlement_type | 1 | 27.52 | 14.81 | 0.18 | 0.20 | 34.49 | 58.83 | 6.93 | 6.92 | 0.19 | 0.10 | 17.12 | 23.37 |
| cass_dist:cass_summary | 11 | 488.61 | 485.16 | 120.84 | 204.27 | 339.17 | 215.51 | 425.76 | 523.83 | 127.91 | 241.04 | 577.54 | 441.96 |
| cass_type:cass_dist | 3 | 16.98 | 18.55 | 34.16 | 111.02 | 795.11 | 728.77 | 29.59 | 28.49 | 50.59 | 138.86 | 681.69 | 661.31 |
| cass_type:cass_summary | 6 | 31.85 | 56.00 | 69.87 | 121.79 | 151.68 | 63.07 | 51.59 | 108.45 | 126.42 | 147.19 | 338.33 | 104.16 |
| population_type:population_dist | 3 | 135.05 | 191.29 | 324.67 | 192.40 | 125.80 | 390.00 | 108.88 | 118.88 | 195.36 | 98.10 | 99.27 | 266.63 |
| population_type:population_summary | 6 | 53.53 | 61.30 | 252.14 | 274.29 | 71.41 | 192.03 | 35.85 | 47.69 | 212.48 | 199.09 | 39.81 | 98.18 |
| settlement_type:settlement_dist | 2 | 916.27 | 865.56 | 38.97 | 323.10 | 101.60 | 470.08 | 284.74 | 426.09 | 5.23 | 167.57 | 16.89 | 88.35 |
| modeltype:cass_type | 1 | 50.53 | 264.57 | 1043.23 | 811.80 | 145.96 | 527.51 | 459.01 | 598.58 | 1246.54 | 840.24 | 116.92 | 232.62 |
| modeltype:population_type | 1 | 0.18 | 6.38 | 33.41 | 62.38 | 34.05 | 26.62 | 2.69 | 23.28 | 33.15 | 65.61 | 34.98 | 25.89 |
| modeltype:settlement_type | 1 | 221.78 | 319.98 | 211.75 | 184.11 | 50.94 | 63.86 | 285.01 | 303.77 | 260.14 | 180.13 | 109.39 | 182.97 |
| modeltype:cass_dist | 3 | 272.90 | 236.80 | 252.00 | 225.33 | 601.27 | 946.36 | 301.86 | 358.90 | 392.93 | 197.48 | 307.13 | 965.18 |
| modeltype:cass_summary | 6 | 342.38 | 212.14 | 710.28 | 687.13 | 496.95 | 275.41 | 657.96 | 391.53 | 948.80 | 691.03 | 480.67 | 414.23 |
| cass_type:population_type:settlement_type | 1 | 0.22 | 0.12 | 0.37 | 0.84 | 0.11 | 0.08 | 0.29 | 0.15 | 0.72 | 1.32 | 0.02 | 0.01 |
| cass_type:cass_dist:cass_summary | 11 | 35.16 | 36.91 | 25.39 | 48.92 | 358.43 | 212.11 | 47.73 | 60.49 | 31.94 | 64.38 | 353.38 | 270.13 |
| population_type:population_dist:population_summary | 10 | 105.80 | 133.70 | 182.11 | 227.28 | 21.45 | 68.62 | 93.79 | 125.73 | 109.82 | 155.05 | 13.05 | 36.48 |
| modeltype:cass_type:population_type | 1 | 0.29 | 0.67 | 1.81 | 1.02 | 0.96 | 0.81 | 1.52 | 4.37 | 3.64 | 1.49 | 2.19 | 3.41 |
| modeltype:cass_type:settlement_type | 1 | 17.87 | 16.18 | 32.93 | 19.20 | 26.85 | 58.02 | 12.56 | 6.09 | 21.50 | 11.28 | 17.55 | 32.95 |
| modeltype:population_type:settlement_type | 1 | 2.02 | 0.01 | 0.01 | 0.89 | 9.18 | 4.63 | 0.01 | 2.15 | 0.29 | 2.94 | 5.14 | 2.88 |
| modeltype:cass_dist:cass_summary | 11 | 236.59 | 156.48 | 116.83 | 177.23 | 543.30 | 361.12 | 275.63 | 211.27 | 140.64 | 201.73 | 917.69 | 712.30 |
| modeltype:cass_type:cass_dist | 3 | 46.32 | 94.61 | 50.02 | 138.33 | 1087.82 | 837.69 | 77.58 | 127.81 | 70.02 | 165.08 | 935.06 | 788.93 |
| modeltype:cass_type:cass_summary | 6 | 15.69 | 48.50 | 96.10 | 193.78 | 292.18 | 124.37 | 70.29 | 85.73 | 197.82 | 243.81 | 582.04 | 190.05 |
| modeltype:population_type:population_dist | 3 | 94.91 | 108.59 | 468.87 | 191.61 | 49.39 | 157.19 | 148.52 | 96.21 | 309.47 | 112.63 | 44.11 | 122.20 |
| modeltype:population_type:population_summary | 6 | 63.63 | 110.01 | 349.11 | 350.94 | 46.75 | 112.14 | 92.86 | 146.25 | 354.78 | 336.00 | 31.83 | 75.25 |
| modeltype:settlement_type:settlement_dist | 2 | 211.52 | 343.79 | 37.86 | 117.40 | 44.13 | 92.22 | 86.49 | 183.72 | 87.55 | 164.62 | 42.10 | 62.30 |
| modeltype:cass_type:population_type:settlement_type | 1 | 0.03 | 0.00 | 0.14 | 0.06 | 0.00 | 0.39 | 0.06 | 0.08 | 0.64 | 0.36 | 0.03 | 0.33 |
| modeltype:cass_type:cass_dist:cass_summary | 11 | 41.41 | 32.33 | 18.64 | 10.74 | 556.43 | 331.25 | 56.86 | 37.15 | 23.49 | 9.34 | 551.68 | 420.97 |
| modeltype:population_type:population_dist:population_summary | 10 | 30.54 | 51.76 | 114.32 | 26.75 | 8.78 | 13.21 | 45.03 | 43.63 | 77.37 | 25.84 | 2.67 | 6.15 |

**S5 Table.** Type-II ANOVA tables for the AIC and adjusted R^2^ extracted from all individual models fitted to Uganda. Only F-statistics are shown, with 31026 residual degrees of freedom, most terms greater than 1 are significant.

|  |  | **Total cassava area** | | **Total monoculture area** | | **Total intercropped area** | | **Total cassava area (weighted)** | | **Total monoculture area (weighted)** | | **Total intercropped area (weighted)** | |
| --- | --- | --- | --- | --- | --- | --- | --- | --- | --- | --- | --- | --- | --- |
| **Term** | **DF** | **F (AIC)** | **F (adj. R2)** | **F (AIC)** | **F (adj. R2)** | **F (AIC)** | **F (adj. R2)** | **F (AIC)** | **F (adj. R2)** | **F (AIC)** | **F (adj. R2)** | **F (AIC)** | **F (adj. R2)** |
| modeltype | 1 | 9746.59 | 21637.71 | 3883.32 | 7134.99 | 16627.41 | 15478.72 | 8318.54 | 18356.28 | 3686.50 | 7692.37 | 16299.79 | 15788.41 |
| cass_type | 1 | 148.29 | 379.04 | 1.72 | 0.01 | 3.95 | 15.67 | 139.35 | 290.76 | 1.77 | 3.61 | 7.45 | 20.35 |
| population_type | 1 | 132.79 | 97.04 | 2.85 | 134.19 | 83.50 | 279.23 | 158.31 | 86.74 | 4.74 | 136.37 | 83.13 | 279.42 |
| settlement_type | 1 | 552.83 | 315.49 | 57.07 | 2468.21 | 3380.02 | 6662.35 | 992.23 | 679.83 | 69.45 | 2596.83 | 3333.40 | 6488.36 |
| cass_dist | 3 | 1265.49 | 1344.58 | 9106.51 | 2701.69 | 2963.41 | 106.17 | 1620.02 | 1735.99 | 12295.26 | 2362.58 | 2989.16 | 111.17 |
| cass_summary | 6 | 162.13 | 544.15 | 180.25 | 101.79 | 14.58 | 13.84 | 141.71 | 536.66 | 172.95 | 94.29 | 14.36 | 11.69 |
| cass_type:population_type | 1 | 0.05 | 0.41 | 0.03 | 0.48 | 0.45 | 2.27 | 0.01 | 2.55 | 0.00 | 0.29 | 0.32 | 2.07 |
| cass_type:settlement_type | 1 | 6.73 | 14.97 | 10.90 | 18.66 | 0.25 | 2.59 | 11.83 | 21.42 | 12.02 | 25.49 | 0.13 | 2.07 |
| population_type:settlement_type | 1 | 0.67 | 0.83 | 74.45 | 476.17 | 14.29 | 111.11 | 9.66 | 11.14 | 96.96 | 474.79 | 13.64 | 106.33 |
| cass_dist:cass_summary | 11 | 267.13 | 331.19 | 585.31 | 519.46 | 11.31 | 9.42 | 441.99 | 492.60 | 641.46 | 522.96 | 9.11 | 8.71 |
| cass_type:cass_dist | 3 | 8.12 | 18.00 | 18.87 | 6.24 | 2.59 | 0.45 | 6.78 | 8.78 | 50.78 | 12.76 | 1.72 | 0.46 |
| cass_type:cass_summary | 6 | 63.99 | 41.88 | 81.03 | 72.62 | 56.24 | 29.80 | 92.66 | 57.23 | 121.79 | 98.40 | 47.92 | 27.87 |
| population_type:population_dist | 3 | 274.58 | 382.89 | 104.00 | 241.06 | 356.11 | 739.99 | 203.76 | 350.68 | 111.91 | 216.96 | 328.63 | 730.65 |
| population_type:population_summary | 6 | 190.67 | 287.67 | 109.17 | 360.62 | 308.08 | 360.62 | 143.13 | 235.72 | 133.45 | 396.03 | 278.15 | 337.84 |
| settlement_type:settlement_dist | 2 | 101.67 | 251.17 | 18.75 | 78.56 | 45.29 | 514.64 | 155.05 | 365.33 | 15.75 | 42.84 | 72.41 | 484.81 |
| modeltype:cass_type | 1 | 20.97 | 224.39 | 0.02 | 68.72 | 0.01 | 0.01 | 0.28 | 201.68 | 8.70 | 53.70 | 0.10 | 0.14 |
| modeltype:population_type | 1 | 14.34 | 37.78 | 3.29 | 15.56 | 87.75 | 37.01 | 10.40 | 41.38 | 6.51 | 25.04 | 83.39 | 35.33 |
| modeltype:settlement_type | 1 | 225.73 | 478.49 | 1.88 | 5.33 | 773.81 | 16.19 | 158.75 | 394.75 | 0.34 | 17.21 | 789.56 | 18.60 |
| modeltype:cass_dist | 3 | 1032.07 | 795.30 | 1707.58 | 1413.08 | 24.20 | 21.68 | 1231.09 | 857.47 | 1728.43 | 1213.44 | 26.84 | 23.19 |
| modeltype:cass_summary | 6 | 173.03 | 320.47 | 383.73 | 295.81 | 18.17 | 12.15 | 243.20 | 372.07 | 406.50 | 287.59 | 15.99 | 11.04 |
| cass_type:population_type:settlement_type | 1 | 1.37 | 2.91 | 2.28 | 6.96 | 0.00 | 0.51 | 3.02 | 4.42 | 2.84 | 7.26 | 0.00 | 0.53 |
| cass_type:cass_dist:cass_summary | 11 | 98.86 | 73.48 | 90.83 | 55.64 | 4.17 | 3.46 | 124.85 | 65.11 | 105.70 | 43.71 | 3.90 | 3.50 |
| population_type:population_dist:population_summary | 10 | 94.02 | 93.23 | 30.87 | 64.76 | 105.46 | 49.21 | 65.37 | 91.07 | 29.75 | 56.55 | 84.78 | 47.48 |
| modeltype:cass_type:population_type | 1 | 3.26 | 3.93 | 8.48 | 11.81 | 1.30 | 2.48 | 4.77 | 5.35 | 10.44 | 17.06 | 1.17 | 2.58 |
| modeltype:cass_type:settlement_type | 1 | 4.27 | 12.01 | 3.00 | 2.99 | 0.30 | 2.16 | 4.26 | 22.44 | 1.87 | 3.21 | 0.27 | 1.51 |
| modeltype:population_type:settlement_type | 1 | 0.02 | 0.09 | 0.52 | 0.25 | 0.43 | 0.39 | 0.12 | 0.06 | 0.01 | 0.76 | 0.43 | 0.59 |
| modeltype:cass_dist:cass_summary | 11 | 215.68 | 130.36 | 479.10 | 223.54 | 15.27 | 10.58 | 314.42 | 150.26 | 488.83 | 217.40 | 11.81 | 10.15 |
| modeltype:cass_type:cass_dist | 3 | 10.53 | 11.52 | 41.33 | 33.56 | 7.75 | 3.17 | 22.42 | 6.56 | 61.33 | 17.02 | 6.07 | 3.89 |
| modeltype:cass_type:cass_summary | 6 | 169.88 | 241.58 | 86.25 | 41.16 | 64.33 | 53.90 | 164.40 | 134.22 | 119.55 | 48.25 | 56.73 | 53.66 |
| modeltype:population_type:population_dist | 3 | 63.85 | 52.79 | 33.16 | 26.33 | 405.97 | 181.27 | 18.78 | 17.22 | 29.98 | 20.54 | 351.61 | 169.83 |
| modeltype:population_type:population_summary | 6 | 151.31 | 109.19 | 90.11 | 267.38 | 242.67 | 93.95 | 80.37 | 95.18 | 105.76 | 354.27 | 207.20 | 83.49 |
| modeltype:settlement_type:settlement_dist | 2 | 22.51 | 11.67 | 117.89 | 223.32 | 736.04 | 433.76 | 54.46 | 44.89 | 150.33 | 226.87 | 817.99 | 475.67 |
| modeltype:cass_type:population_type:settlement_type | 1 | 0.48 | 1.03 | 0.15 | 0.07 | 0.26 | 0.87 | 0.12 | 1.03 | 0.03 | 0.31 | 0.23 | 0.94 |
| modeltype:cass_type:cass_dist:cass_summary | 11 | 120.11 | 58.09 | 101.25 | 49.54 | 5.25 | 4.19 | 137.48 | 41.34 | 113.09 | 35.75 | 4.64 | 3.90 |
| modeltype:population_type:population_dist:population_summary | 10 | 85.09 | 43.29 | 21.16 | 79.96 | 104.75 | 33.57 | 39.31 | 39.53 | 19.34 | 68.49 | 72.78 | 27.48 |
