# Supplementary material for: Validating a cassava production spatial disaggregation model in sub-Saharan Africa
Source: PLoS One. 2024 Nov 5;19(11):e0312734. doi: 10.1371/journal.pone.0312734 (PMC11537372; doi:10.1371/journal.pone.0312734)
Supplement: Attachment — Submitted filename: review comments and suggestions.doc [file pone.0312734.s003.doc]

**Validating a cassava spatial disaggregation model in sub-Saharan Africa**

|  | **Comments and suggestions** |
| --- | --- |
| **Abstract** | On line 31, include the future beneficiaries of this research  In this subheading, answer this questions why, what, how and what for  mention the importance of these studies |
| **Introduction** | Line 35, include the reference on the end of period and do this to whole document  from line 41 to 44, unbold and include the information related with cassava yield and the most cassava countries producers.  Line 49, include the reference and mention about studies/ researches related with this subject, describe the methods/ modells and explain why the authors are using this methods, I mean the advantages  line 64, why are you using references on the objective?  Mention the importance of the information of distribution of cassava cultivation, of non-urban population  mention something about satellites measurement difficult |
| **Material and methods** | Line 72, there some error, please check and correct, do the same within whole manuscript  line 73, include the reference  I suggest to divide this subheading (locations of the survey, climate and soil, socio-geografic data, farm level, remoting sensing, response variables, statistical analysis, prodution and harvest, administrative units, missing data, spacial and temporal disaggregation, data record ) in order to facilitate the explanation to the readers and avoid misunderstood.  lines 105 and 106, I suggest to include the comma instead and N, and K  line 111 to 113, merge it  line 117, rewrite  line 122, rewrite 1 kmx1Km  line 132, is it comparison or correlation  line 155 to line 163, rewrite and focus on the approach, methods that you used to collect the data  line 165, merge it  on table 3, check the last formula, is that correct?  Line 199 to 201 include the references of AIC and BIC |
| **Results** | Line 239, include the reference  line 243 to 245, the total is not 100% locations, could please check it  line 252 and 253, the total is not correct, please check it  line 275, is table 3 or 4, check it, How can we use this information? I notice thar correlation is between response variables. Clarify it,  line 309, include the Anova or in the supplementary data  line 328, include the values of AIC |
| **Discussion** | Separate this chapter with conclusions  Do not forget to compare your results with others studies |
| **conclusion** | Link or focus on the objectives |
| **References** | After consider all corrections and suggestions, cross-check the references within manuscript |

Moiana, LD

Genetics and Breeding, Dr. Sc

Senior Research at IIAM/Mozambique

Scientific Coordinator at Rcol for Rice-Namacurra/Zambezia
