# Supplementary material for: Validating a cassava production spatial disaggregation model in sub-Saharan Africa
Source: PLoS One. 2024 Nov 5;19(11):e0312734. doi: 10.1371/journal.pone.0312734 (PMC11537372; doi:10.1371/journal.pone.0312734)
Supplement: Attachment — Submitted filename: MapSPAM and Cassava Map reviewers notes.docx [file pone.0312734.s004.docx]

**Reviewer’s Comments: Validating a cassava spatial disaggregation model in sub-Saharan Africa**

Line 64 Provide a brief description of MapSPAM and the required inputs and CassavaMap. Why were they selected? This information could be placed in a paragraph before the objectives.

Lines 70-71 Provide general background information on the locations. How do they differ? You may look at the population size (may be specific to rural), demographics, amount of lands dedicated to agriculture, prevailing climate.

Lines 75-76 What was the proportion of the levels of population densities among the number of surveyed locations in each country?

Line 86 Define “high, medium and low density”.

Line 129 Were the latest versions or data set of CassavaMap and MapSPAM used at the time of writing?

Line 133 Which statistical package was used for the correlation analyses?

Lines 234-242 Bits on field attributes could be placed in a table to describe the locations/countries for easier reading and comparison. Same applies for Lines 255-264.

Line 239 Present means with standard dev. since that was mentioned as calculated in the method. Adjust throughout results section.

Line 251 Were 96 or 87 locations assessed in Uganda?

Lines 279-282 Were the uncertainty in the predicted values presented?

Lines 378-383 not necessary for the discussion, can be placed in the Introduction.

Lines 384-389 Method should not be in the discussion. More discussion of the results is needed. Highlight the responses (results in context) to the objectives of the study. Make reference to recent work using MapSPAM whether in cassava or other crops.

Table 3 keep variable names consistent.

Figure 4 Fig. a Scale on the X axis, “20,000” reported as 2,000. Use of a consistent scale is recommended for country comparisons.

Variable names in most figures should be typed out appropriately.
