# Supplementary material for: Validating a cassava production spatial disaggregation model in sub-Saharan Africa
Source: PLoS One. 2024 Nov 5;19(11):e0312734. doi: 10.1371/journal.pone.0312734 (PMC11537372; doi:10.1371/journal.pone.0312734)
Supplement: Attachment — Submitted filename: Response to reviewers.docx [file pone.0312734.s005.docx]

**Response to reviews**

We thank the reviewers and editors for their time in considering our manuscript. In the response below, the comments of the editor and each reviewer are addressed. We begin with the editor, reviewer 1, and finally addressing reviewers’ 2 comments.

The editor and the reviewers’ comments are shown in italics and in blue. The line numbers provided in the reviewers’ comments refer to the originally submitted manuscript.

Our responses to each comment are immediately below and, unless otherwise stated, the line and equation numbers refer to the clean revised manuscript.

**Editor**

1. *I recommend that you provide a description the programs used in the introduction and provide more information on why these programs were used.*

This has been added in lines 106-110

2. *Population density is subjectively used here. Can you provide more information as to how high, medium, and low-density population levels were defined? More details are needed in the methods section on the locations used as suggested. For example, details on the population, area under agriculture production, and climate are required to properly define the study locations.*

The way we used the term “density” is referring to the cassava cultivation density, not the human population. The way we classified density is now made explicit in lines 137-139. In terms of human population, we specify in lines 125-126 the types of areas surveyed. We also added a couple of lines (111-112) on the climate of the regions explored in this study.

3. *In Table 3 please keep variable names consistent.*

This has been fixed (Table 5).

4. *There seems to be a conflict with the information in Figure 4. Fig. a Scale on the X axis, “20,000” reported as 2,000.*

This has been corrected.

5. *Use of a consistent scale for country comparisons.*

This has been corrected.

6. *Variable names in most figures should be typed out.*

These have been added and corrected as necessary.

7. *Please ensure that your manuscript meets PLOS ONE's style requirements, including those for file naming.*

We have ensured that the style and the file naming are correct.

8. *In your Methods section, please provide additional information regarding the permits you obtained for the work. Please ensure you have included the full name of the authority that approved the field site access and, if no permits were required, a brief statement explaining why.*

We have added a couple of lines (124-125) specifying how did we get permission to access the sites.

9. *Please note that PLOS ONE has specific guidelines on code sharing for submissions in which author-generated code underpins the findings in the manuscript. In these cases, all author-generated code must be made available without restrictions upon publication of the work.*

This has been made available (see the Data Availability Statement)

10. *Please include a complete copy of PLOS’ questionnaire on inclusivity in global research in your revised manuscript. Our policy for research in this area aims to improve transparency in the reporting of research performed outside of researchers’ own country or community. Please upload a completed version of your questionnaire as Supporting Information when you resubmit your manuscript.*

This is included as Supporting Information

11. *When completing the data availability statement of the submission form, you indicated that you will make your data available on acceptance. We strongly recommend all authors decide on a data sharing plan before acceptance, as the process can be lengthy and hold up publication timelines.*

All the data and code needed to reproduce the analyses of our manuscript have been published and are available at the links provided in the Data Availability Statement

12. *We note that [Figure(s) 1, 2, 6a and b, 7a and b in your submission contain [map/satellite] images which may be copyrighted. All PLOS content is published under the Creative Commons Attribution License (CC BY 4.0), which means that the manuscript, images, and Supporting Information files will be freely available online, and any third party is permitted to access, download, copy, distribute, and use these materials in any way, even commercially, with proper attribution.*

We have removed Fig 2 as we were unable to get the basemap copyright. The rest of the figures have been checked and referenced accordingly.

**Reviewer 1:**

**Abstract**

1. *On line 31, include the future beneficiaries of this research. In this subheading, answer this questions why, what, how and what for mention the importance of these studies.*

Done. We have included a paragraph outlining the usefulness and the future beneficiaries of this research from lines 40-45. Some words have been removed earlier to ensure the abstract is still within the word limit.

**Introduction**

2. *Line 35, include the reference on the end of period and do this to whole document.*

Agreed. We have done this in this instance (line 48) and many others (e.g. line 71), unless the reference needed to be next to the information to make it clear what it was referring to.

3. *from line 41 to 44, unbold and include the information related with cassava yield and the most cassava countries producers.*

Thank you, we have changed the font colour throughout the text, as some places it was different in different paragraphs. There is already information on yield from line 52 and which countries are the greatest producers, so we haven’t added any additional information.

4. *Line 49, include the reference and mention about studies/ researches related with this subject, describe the methods/ models and explain why the authors are using this methods, I mean the advantages.*

Line 49 (now line 63) states that there is a lack of verified models describing the distribution and density of cassava cultivation. We reference the only two existing models of cassava cultivation in Africa, which are the Spatial Production Allocation Model MapSPAM and CassavaMap, mentioned and referenced on lines 84-87. We are not sure what the reviewer would like further.

5. *line 64, why are you using references on the objective?*

The references have been removed from the objectives, and the disaggregation models referenced in the previous paragraph (line 81), where the disaggregation models have been explained in a bit more detail.

6. *Mention the importance of the information of distribution of cassava cultivation, of non-urban population.*

We have added information on the consequences of pests and pathogens in leading to yield losses (lines 68-70).

7. *mention something about satellites measurement difficult.*

This is addressed in lines 73-75.

**Material and methods**

8. *Line 72, there some error, please check and correct, do the same within whole manuscript.*

Thank you. It was a malfunctioning reference to Figure 1. This has now been corrected.

9. *line 73, include the reference.*

This has been added (line 108).

10. *I suggest to divide this subheading (locations of the survey, climate and soil, socio-geographic data, farm level, remoting sensing, response variables, statistical analysis, production and harvest, administrative units, missing data, spatial and temporal disaggregation, data record) in order to facilitate the explanation to the readers and avoid misunderstood.*

We have added additional sub-headings to make the structure clearer.

11. *lines 105 and 106, I suggest to include the comma instead and N, and K*

Agreed. We added a couple of semicolons to make clearer the separation between each term. We kept $\alpha$ and $M$ in the same clause as they belong to the same term in the equation, as well as $\beta$ and $M$, and $\gamma$ and $K$ (lines 153-156).

12. *line 111 to 113, merge it.*

We thank the reviewer for pointing out this error. This is, again, a problem with a malfunctioning reference (in this case to Table 1). We have removed all the hyperlinked references and rewritten them as plain text.

13. *line 117, rewrite*

See comment to point 24.

14. *line 122, rewrite 1 kmx1Km*

This text has been moved and replaced but we have made sure to correct this type of errors.

15. *line 132, is it comparison or correlation.*

We have rephrased this sentence to read “We compared…” (line 170)

16. *line 155 to line 163, rewrite and focus on the approach, methods that you used to collect the data.*

To clarify the text in this section we rephrased some of the text (lines 188-192).

17. *line 165, merge it.*

Thank you. This has been fixed.

18. *On table 3, check the last formula, is that correct?*

Yes, this is correct. As stated in line 227, the first type of regression model explored was a simple linear regression model. We added a line in the caption of Table 3 to clarify this.

19. *Line 199 to 201 include the references of AIC and BIC*

We have added the definitions for AIC and BIC in lines 240-245.

**Results**

20. *Line 239, include the reference.*

Unfortunately, we do not understand this request. There is nothing to be referenced here as we are only talking about the characteristics of cassava cultivation from the survey.

21. *line 243 to 245, the total is not 100% locations, could please check it.*

The text is correct. We are not measuring the number of locations but the ratio of intercropped:monoculture fields in each location. Across the 52 locations, the locations in complete monoculture were 27%, in complete intercropping were 15% and in a 50:50 split there were 23% of the locations. The rest of the locations were somewhere in between total monoculture and total intercropping and a 50:50 split (e.g. a 30:70 split or a 80:20 split). We have added a sentence in lines 289-291 to make this more explicit.

22. *line 252 and 253, the total is not correct, please check it.*

The text is correct. At each survey site, there may be several fields, which means that in each location you can have either monoculture, intercropped, individual plants or a combination of these three. Therefore, each location can contain fields with one or more cultivation methods.

23. *line 275, is table 3 or 4, check it. How can we use this information? I notice that correlation is between response variables. Clarify it.*

It is now Table 5. The referencing hyperlink was again malfunctioning. This is now corrected. As stated in lines 321-322 this table indicates that no detectable relationships were observed between predicted cassava production at the surveyed point locations and the observed cassava area in either Côte d'Ivoire or Uganda.

24. *line 309, include the Anova in the supplementary data.*

These are now included in the supplementary data as S2 and S3 Tables and referenced in lines 365-366 of the main text.

25. *line 328, include the values of AIC.*

These have been added in lines 380-382.

**Discussion**

26. *Separate this chapter with conclusions.*

The journal does not require to separate the discussion from the conclusions. We believe that the manuscript flows better if we keep a single section for both, so we kept in that way.

27. *Do not forget to compare your results with others studies.*

These are discussed in the first paragraph of the “Discussion and conclusions” section (lines 416-426).

**Conclusion**

28. *Link or focus on the objectives.*

Although this was implicit across the discussion, we addressed the reviewers' request by adding a few lines (434-435, 438-440) to make the objectives and the discussion link clearer. We also extended some sections of the discussion to make links between our hypotheses and results clearer.

**References**

29. *After consider[ing] all corrections and suggestions, cross-check the references within manuscript*

We have cross-checked that all the references are correct.

**Reviewer 2:**

1. *The data presented in the manuscript supports the conclusion drawn. However, there are some areas for improvement especially in the Discussion section.*

We thank the reviewer for this comment. Accordingly, we have extended the discussion making more explicit links with our findings and what they may mean in a wider context.

2. *Where statistical analyses were performed, measures of variability need to be presented as well as errors for predicted and measured values in the models used.*

We agree with the reviewer that, in general, measures of variability should be included when presenting statistical results. However, we are not quite sure where the reviewer specifically thinks this should be included in the manuscript. We have attempted to include additional measures of variability where we can. Specifically, we have added some additional summary statistics in Tables 3 and 4 (namely lower and upper quartiles) of cultivation area and field size.

For the remaining statistical analyses, we do not think it necessary to include measures of variability as we typically present model outputs as summaries. For example, Fig 4 shows the boxplots of predictions over all locations and therefore we do not present individual point predictions (and associated uncertainty). Similarly, for the regression analysis of Fig 7, we present boxplots of the model predictions summarized over the different combinations of covariates and again do not present individual predictions.

3. *Line 64 Provide a brief description of MapSPAM and the required inputs and CassavaMap. Why were they selected? This information could be placed in a paragraph before the objectives.*

This information is now added in lines 85-107 in the introduction.

4. *Lines 70-71 Provide general background information on the locations. How do they differ? You may look at the population size (may be specific to rural), demographics, amount of lands dedicated to agriculture, prevailing climate.*

This information is now added in line 134-139.

5. *Lines 75-76 What was the proportion of the levels of population densities among the number of surveyed locations in each country?*

We don’t fully understand this question. The areas surveyed were chosen at random along major motorable roads in each country which meant that the survey locations included rural, suburban, and urban areas, but no record was taken about the type of environment where each building was (although specifics of some buildings and their surroundings can be found in the original dataset <https://doi.org/10.6084/m9.figshare.23657391.v1>)

6. *Line 86 Define “high, medium and low density”.*

We have now defined these concepts in line 137-139.

7. *Line 129 Were the latest versions or data set of CassavaMap and MapSPAM used at the time of writing?*

Our analyses were developed using the most recent version of MapSPAM and CassavaMap.

8. *Line 133 Which statistical package was used for the correlation analyses?*

We used the using the R package ggcorrplot. This has now been added in lines 171-172

9. *Lines 234-242 Field attributes could be placed in a table to describe the locations/countries for easier reading and comparison. Same applies for Lines 255-264.*

We thank the reviewer for this suggestion. Tables 3 and 4 have now been added to the document to summarise the field attributes of the locations surveyed.

10. *Line 239 Present means with standard dev. since that was mentioned as calculated in the method. Adjust throughout results section.*

In lines 283-284 (previously 239-240), we simply report the percentage of locations that have only monoculture fields, only intercropped fields or 50:50 split. There is no variability to calculate. The reference to standard deviation in the methods refers to one of the summary statistics we calculated from the buffered data layers and is only relevant to the regression models reported later.

11. *Line 251 Were 96 or 87 locations assessed in Uganda?*

There were 87 locations assessed in Uganda. We have corrected this throughout the text.

12. *Lines 279-282 Were the uncertainty in the predicted values presented?*

No. Measures of uncertainty for production model predictions (from CassavaMap and MapSPAM) were not available.

13. *Lines 378-383 not necessary for the discussion, can be placed in the Introduction.*

We agree with the reviewer that the way these lines were written did not belonged in the discussion. However, another anonymous reviewer requested that the objectives were linked to our discussion, so we rewrote this section to make it the link clearer.

14. *Lines 384-389 Method should not be in the discussion. More discussion of the results is needed. Highlight the responses (results in context) to the objectives of the study. Make reference to recent work using MapSPAM whether in cassava or other crops.*

We agree with the reviewer. Some of these lines have now been deleted and the rest has been put into the discussion context. Additionally, we added some text putting the objectives in the context of the discussion (434-439) and added some text discussing the results of our analyses (lines 448-453, 469-488). We referred to recent work on MapSPAM being used more recently in lines 89-97.

15. *Table 3 keep variable names consistent.*

This has been corrected.

16. *Figure 4 Fig. a Scale on the X axis, “20,000” reported as 2,000. Use of a consistent scale is recommended for country comparisons.*

This has been amended so that the X axis scale reads correctly.

17. *Variable names in most figures should be typed out appropriately.*

We appreciate this observation from the reviewer. All the figures needing units and changes have been modified to represent accurately variables and units.
